# Zebrafish obesogenic test identifies anti‐adipogenic fraction in *Moringa oreifera* leaf extracts

**DOI:** 10.1002/fsn3.2758

**Published:** 2022-02-11

**Authors:** Izumi Matsuoka, Kanae Hata, Hirotaka Katsuzaki, Hiroko Nakayama, Liqing Zang, Mizuho Ota, Youngil Kim, Djong‐Chi Chu, Lekh Raj Juneja, Norihiro Nishimura, Yasuhito Shimada

**Affiliations:** ^1^ 12946 Graduate School of Regional Innovation Studies Mie University Tsu Japan; ^2^ 12946 Graduate School of Bioresources Mie University Tsu Japan; ^3^ Mie University Zebrafish Drug Screening Center Tsu Japan; ^4^ Rohto Pharmaceutical Co., Ltd. Osaka Japan; ^5^ Department of Bioinformatics Mie University Advanced Science Research Promotion Center Tsu Japan; ^6^ Department of Integrative Pharmacology Mie University Graduate School of Medicine Tsu Japan

**Keywords:** diabetes, dyslipidemia, herbal medicine, natural products, visceral obesity

## Abstract

The zebrafish obesogenic test (ZOT) is a powerful tool for identifying anti‐adipogenic compounds for in vivo screening. In our previous study, we found that *Moringa oleifera* (MO) leaf powder suppressed the accumulation of visceral adipose tissue (VAT) in ZOT. MO demonstrates a wide range of pharmacological effects; however, little is known about its functional constituents. To identify the anti‐adipogenic components of MO leaves, we prepared extracts using different extraction methods and tested the obtained extracts and fractions using ZOT. We found that the dichloromethane extract and its hexane:EtOAc = 8:2 fraction reduced VAT accumulation in young zebrafish fed a high‐fat diet. We also performed gene expression analysis in the zebrafish VAT and found that CCAAT/enhancer‐binding protein beta and CCAAT/enhancer‐binding protein delta (associated with early stages of adipogenesis) gene expression was downregulated after fraction 2 administration. We identified a new MO fraction that suppressed VAT accumulation by inhibiting early adipogenesis using the ZOT. Phenotype‐driven zebrafish screening is a reasonable strategy for identifying bioactive components in natural products.

## Graphical abstract

We performed zebrafish obesogenic test to identify anti‐adipogenic fraction in Moringa oleifera leaf. Phenotype‐driven zebrafish screening can be a reasonable strategy for identifying bioactive components in natural products. Using zebrafish obesogenic test, we identified that the dichloromethane extract of *Moringa oleifera* leaf and its subfraction (Fr. 2) reduced VAT accumulation in young zebrafish. In the zebrafish VAT, expression of early adipogenesis markers, *cebpb* and *cebpd*, was significantly (*p* < .05) decreased by Fr. 2, as was the expression of the late differentiation marker *cebpa*. We also confirmed that the subfractions of Fr. 2 also suppressed adipogenesis in ZOT and mouse 3T3‐L1 preadipocytes.

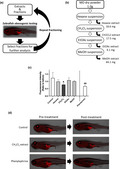

## 1 INTRODUCTION

Excessive accumulation of visceral adipose tissue (VAT) leads to metabolic syndromes, including type 2 diabetes, hypertension, dyslipidemia, and atherosclerosis, which shorten the lifespan and reduce the quality of life. Dieting and physical exercise are the main approaches in reducing adiposity, although many obese people receive anti‐obesity medications. Currently available anti‐obesity medications can cause severe side effects. For example, the CB1 receptor agonist rimonabant may cause psychiatric diseases, including depression and insomnia (Christensen et al., 2007), whereas the sympathomimetic agent phentermine demonstrates cardiovascular, such as hypertension, and psychiatric adverse effects (Kang & Park, 2012). Thus, natural product‐based obesity treatment with fewer adverse effects would lead to a considerable advancement of obesity management.

The zebrafish (*Danio rerio*) is an important model organism for studying human diseases, with a high degree of similarity to humans in terms of organ structure and genome sequence. It has been increasingly utilized in drug discovery owing to its compatibility with in vivo imaging, harmonization with animal rights management methods, and optimization of methods for chemical screening (Lieschke & Currie, 2007; MacRae & Peterson, 2015). The similarity of lipid metabolism and adipogenesis between zebrafish and mammals has been demonstrated. Overfeeding‐ or high‐fat diet (HFD)‐induced obesity in zebrafish yields symptoms similar to those in humans, such as hypertriglyceridemia, hepatosteatosis, and visceral adiposity, and share pathophysiological pathways of obesity common in mammals (Minchin & Rawls, 2017; Shimada, Kuninaga, et al., 2014; Zang et al., 2018). For example, important genes involved in white adipose tissue development, namely, CCAAT/enhancer‐binding protein beta (*cebpb*), CCAAT/enhancer‐binding protein delta (*cebpd*), peroxisome proliferator‐activated receptor gamma (*pparg*), and CCAAT/enhancer‐binding protein alpha (*cebpa*), are highly conserved in their amino acid sequences and functions between zebrafish and mammals (Imrie & Sadler, 2010; Shimada et al., 2015).

We previously developed a novel diet‐induced adult zebrafish obesity model (Oka et al., 2010) and discovered several natural products with lipid‐lowering and visceral adipose‐reducing properties (Hiramitsu et al., 2014; Nakayama et al., 2018; Shimada et al., 2014; Tainaka et al., 2011; Zang et al., 2015). However, using adult fish is labor‐intensive, the experimental setup is time‐consuming (more than 4 weeks), and it requires a lot of space (number of tanks) because of the size of the fish tanks (more than 3 cm in body length). However, because of the small size of the young zebrafish (less than 1 cm approximately 1 month post fertilization), they could be bred in six‐well plates. Moreover, young zebrafish have a transparent body wall that enables live imaging of internal organs, such as VAT, by labelling them with Nile red (NR) fluorescent dye. This technique is the basis for the zebrafish obesogenic test (ZOT) (Tingaud‐Sequeira et al., 2011; Zang et al., 2019). We previously screened a natural product library using this ZOT and found that *Moringa oleifera* (MO) leaf powder suppressed VAT accumulation (Nakayama et al., 2020).

MO is a perennial plant native to the southern foothills of the Himalayas in northwestern India and is now widely cultivated in tropical and subtropical regions. Its young fruits and leaves can be eaten, and recently, its medicinal properties have also attracted attention of researchers. It has been reported to have a wide range of therapeutic effects (Bhattacharya et al., 2018), including lowering cholesterol (Almatrafi et al., 2017), regulating blood pressure (Chen et al., 2012), improving immunity, reducing inflammation (Omodanisi et al., 2017), suppressing appetite (Ahmad et al., 2018), and controlling blood sugar (Mbikay, 2012).

A limited number of MO‐derived anti‐adipogenic molecules have been identified in mouse 3T3‐L1 adipocytes (Balakrishnan et al., 2018; Xie et al., 2018). Here, we prepared several types of MO extracts and subfractions and performed ZOT to identify the major anti‐obesity components in MO.

## 2 MATERIALS AND METHODS

### 2.1 Extraction and fractionation of MO

**FIGURE 1:**
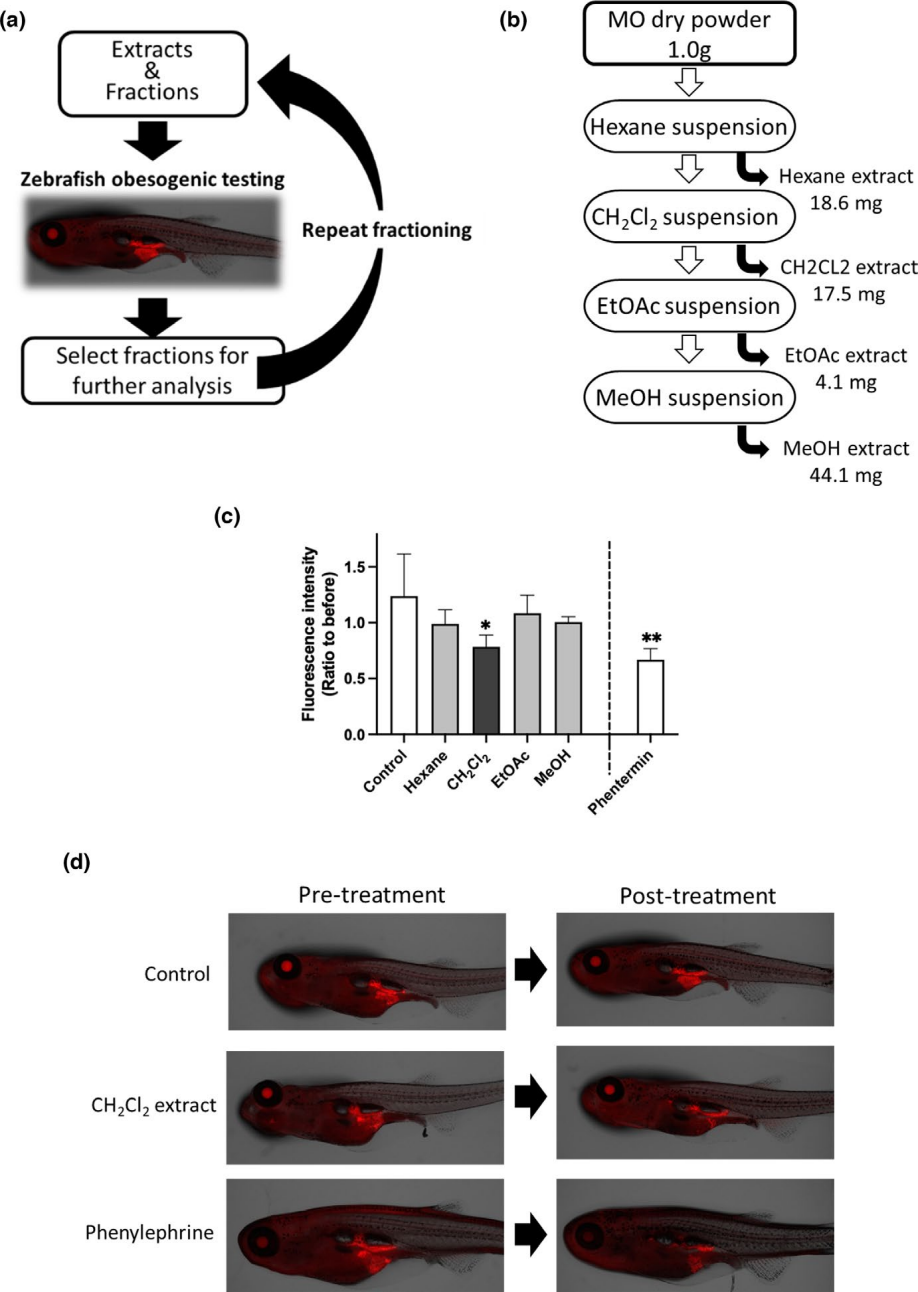
CH_2_Cl_2_ extract reduces total visceral adipose tissue (VAT) in high‐fat diet‐fed zebrafish. (a) Experimental design of this study. (b) Preparation of MO extracts. Extracts were prepared using serial extraction with each solvent. (c) NR fluorescent intensities in the VAT were calculated. The Y‐axis indicates the ratio of NR staining before and after 48‐h treatment with each extract. Data are shown as the means ± standard deviation. *n* = 5, *p < .05 versus control, as calculated by using one‐way ANOVA. Reproducibility test results are displayed in Figure S2. Each extract was administered at 10 μg/ml. Phenylephrine (20 μM) was used as a positive control. (d) Representative images of control‐ and CH_2_Cl_2_ extract‐treated zebrafish

MO leaf powder was prepared at Rohto Pharmaceutical Co., Ltd. Dried and powdered leaves were extracted repeatedly with each solvent at room temperature, as shown in Figure 1b. For further fractionation, the dichloromethane (CH_2_Cl_2_) extract was concentrated, and the resultant residue was loaded onto a silica gel chromatography column (5 cm inner diameter × 15 cm). The captured molecules were eluted with the following solvents: hexane:EtOAc = 10:0, 8:2, 6:4, 4:6, and 0:10 (1 L each) to obtain five fractions, as shown in Figure 3b. Each fraction was concentrated and dried *in vacuo*.

To isolate the active component(s) of fraction 2 (Fr. 2), preparative HPLC was performed. The preparative HPLC conditions were as follows: column, Develosil 100–5 (10φ × 250 mm); flow rate, 3.0 ml/min; elution condition, hexane:IPA = 97:3; and detection, 280 nm. Fr. 2 was separated into six fractions plus washout using preparative HPLC, as shown in Figure 3a,b. Each of the six fractions was assessed using analytical silica gel HPLC to verify that the peaks were separated, as shown in Figure S4. The analytical silica gel HPLC conditions were as follows: column, Develosil 100–5 (4.6φ × 250 mm); flow rate, 1.0 ml/min; elution conditions, hexane:IPA = 97:3; and detection, 280 nm.

### 2.2 Zebrafish strains and maintenance

Zebrafish (AB strain; Zebrafish International Research Center, Eugene, OR, USA) were reared and maintained in our facility according to standard operational guidelines in compliance with international guidelines. Fish were fed GEMMA Micro 75–300 (Skretting, Fontaineles‐Vervins, France) based on their developmental stage and length.

### 2.3 ZOT

The ZOT was performed as previously reported (Tingaud‐Sequeira et al., 2011; Zang et al., 2019) with some modifications. Young zebrafish (4–5 weeks post fertilization, standard length approximately 7–9 mm) were assigned to a control diet or HFD group. For preparing the HFD, 1 g of boiled chicken egg yolk was suspended in 15 ml of water as a stock and stored at −80°C. Five fish were transferred to a six‐well plate in 5 ml of 0.3× Danieau's solution (17.4 mM NaCl, 0.21 mM KCl, 0.12 mM MgSO_4_, 0.18 mM Ca(NO_3_)_2_, and 1.5 mM 4‐(2‐hydroxyethyl)‐1‐piperazinyl‐ethane‐2‐sulfonic acid; pH 7.6) with 10 μg/ml kanamycin (Nacalai Tesque). The HFD group was fed 25 μl of egg yolk suspension twice daily (morning and evening). During HFD feeding, the six‐well plates were shaken at 150 rpm. After the feeding period, zebrafish were stained with 5 μg/ml NR (Tokyo Chemical Industry) in 1% acetone‐H_2_O for 30 min and washed three times with 0.3× Danieau's solution for 10 min. Fish were then anesthetized with 0.003% tricaine (MS222; Sigma‐Aldrich). The NR signal was imaged using a BZ‐X710 fluorescence microscope (TRITC filter; Keyence). NR intensity was quantified using ZF‐Mapper software (Yamamoto et al., 2019). Fish were then treated with extract (10 μg/ml), negative control (0.1% DMSO as a vehicle), or phenylephrine (20 μM) as a positive control (Tingaud‐Sequeira et al., 2011) for 48 h and were again stained with NR to visualize VATs, as shown in Figure S1. Day 3/day 1 ratios were calculated as previously reported (Nakayama et al., 2020).

### 2.4 RNA extraction, cDNA synthesis, and quantitative real‐time PCR (qPCR)

After NR staining, VAT was collected under a fluorescence microscope using an SMZ745T microscope (Nikon) equipped with a fluorescence filter set (NightSea). Total RNA was isolated using an RNeasy Mini Kit (Qiagen). cDNA synthesis was performed using 200 ng of total RNA and a ReverTra Ace qPCR RT Kit (Toyobo). qPCR using cDNA samples was performed with Power SYBR Green Master Mix (Applied Biosystems) and an ABI StepOnePlus Real‐Time PCR System (Applied Biosystems) in accordance with the manufacturer's instructions. The sequences of the forward and reverse primers used for amplification are listed in Table S1. Relative mRNA expression levels were determined using actin beta 1 as an endogenous standard.

### 2.5 3T3‐L1 adipocyte differentiation assay

Mouse 3T3‐L1 preadipocytes were purchased from DS Pharma Biomedical. Preadipocytes were cultured in Dulbecco's modified Eagle's medium–high glucose medium (Gibco, Gaithersburg, MD, USA) supplemented with 10% calf bovine serum (Gibco) and penicillin–streptomycin (Nacalai Tesque) at 37°C in a humidified 5% CO_2_ atmosphere until confluence was reached in a 96‐well plate format. Two days after confluence (day 0), cells were stimulated to differentiate by culturing in adipocyte differentiation medium (ADM; DS Pharma Biomedical) for 3 days. Cells were then maintained in adipocyte maintenance medium (DS Pharma Biomedical) for an additional 4 days. Extracts or fractions (10 μg/ml) were administered from day 0 of adipocyte differentiation. On day 7, intracellular lipid droplets were stained using AdipoRed Assay Reagent (Lonza) according to the manufacturer's instructions. After obtaining images using a BZ‐X710 fluorescence microscope (Keyence), intracellular lipid accumulation was quantified by measuring fluorescence (Ex 485 nm/Em 590 nm) using a Victor2 multilabel plate reader (PerkinElmer).

### 2.6 Statistical analyses

All results are presented as the mean ± standard deviation. Data were analyzed using the Student's *t*‐test or analysis of variance with the Bonferroni–Dunn multiple comparison procedure, depending on the number of comparisons, using GraphPad Prism version 8 (GraphPad Software). Statistical significance was set at *p* < .05.

## 3 RESULTS

### 3.1 Dichloromethane extract of MO reduced VAT accumulation in HFD‐fed zebrafish

This study is conceptually diagrammed in Figure 1a. To identify the bioactive constituents of MO, we first prepared a series of MO extracts using four solvents: hexane, dichloromethane (CH_2_Cl_2_), ethyl acetate (EtOAc), and methanol (MeOH) (Figure 1b). We then performed an adipocyte differentiation assay using the ZOT (Figure 1c) according to previously published methods (Nakayama et al., 2020). One day after feeding the boiled chicken yolk sac (a HFD) to young zebrafish (4–5 weeks post fertilization), total VAT increased, as detected by NR staining. Zebrafish were then treated with MO extracts for 48 h, stained again with NR, and changes in VAT fluorescence intensities were quantified (Figure S1). Before ZOT, we assessed the safety concentrations of these extracts using the zebrafish embryo acute toxicity test (Figure S2) according to the OECD guideline (Busquet et al., 2014) and determined that at 10 μg/ml they did not exhibit toxicity. Among the extracts, the CH_2_Cl_2_ extract significantly (*p* < .05, Figure 1c and Figure S3) reduced VAT accumulation compared to that after the control treatment (0.1% DMSO as a vehicle). Representative images of the NR‐stained fish treated with CH_2_Cl_2_ extracts are shown in Figure 1d.

### 3.2 Subfraction 2 of CH_2_Cl_2_ extract suppressed adipogenesis

**FIGURE 2:**
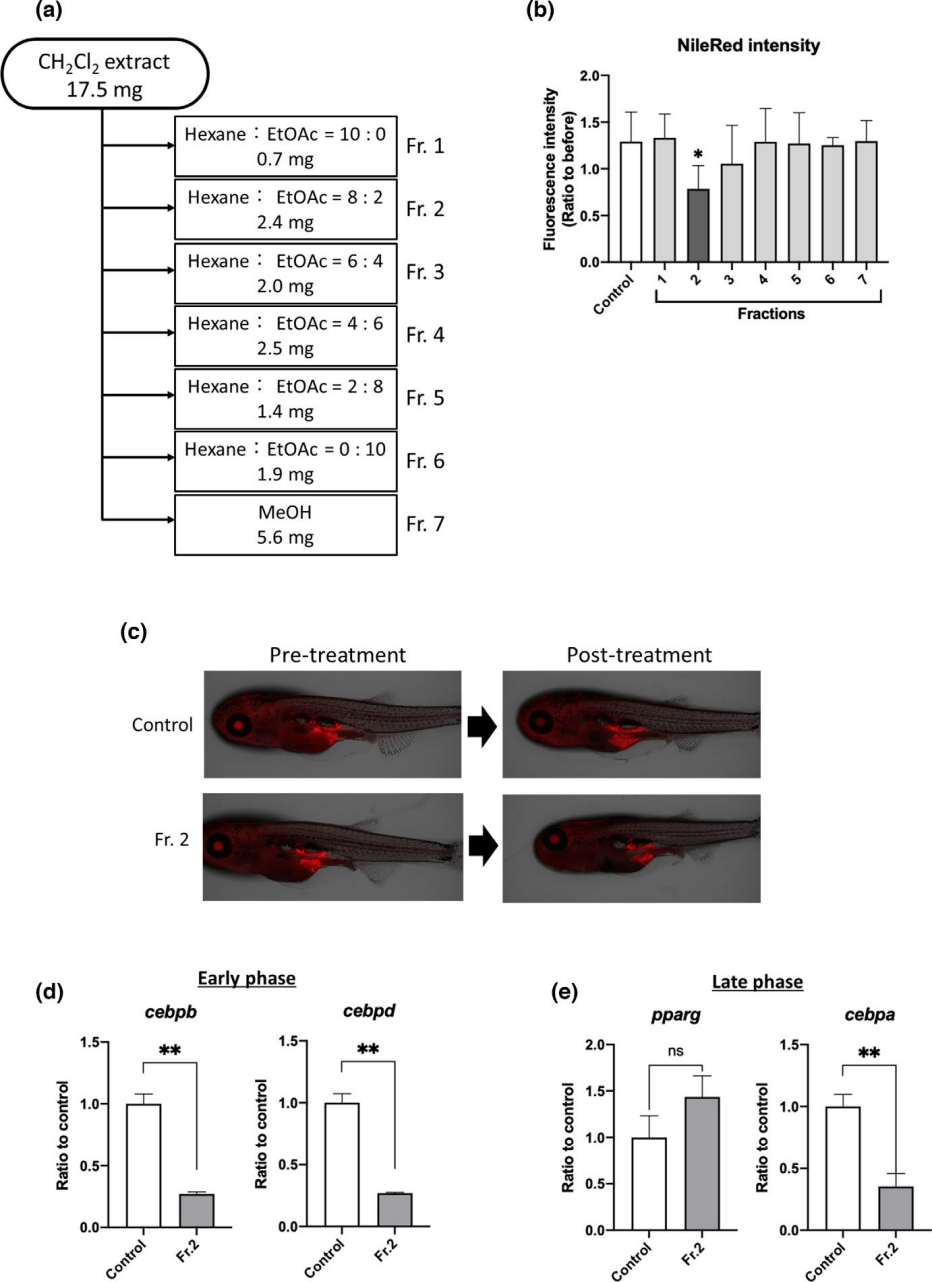
Fraction 2 of CH_2_Cl_2_ extract suppresses adipogenesis in zebrafish VAT. (a) Flow chart showing elution fractions obtained using silica gel chromatography. (b and c) Zebrafish obesogenic test. Fraction 2 (10 μg/ml) reduced the amount of VAT compared to control treatment (0.1% DMSO). Data are shown as the means ± standard deviation. *n* = 5, *p < .05 versus control, as calculated by using one‐way ANOVA. Reproducibility test results are provided in Figure S3. (d and e) qPCR analysis of early adipocyte differentiation markers CCAAT/enhancer‐binding protein beta (*cebpb*) and CCAAT/enhancer‐binding protein delta (*cebpd*; d) and late differentiation markers peroxisome proliferator‐activated receptor gamma (*pparg*) and CCAAT/enhancer‐binding protein alpha (*cebpa*; e). Data are shown as the means ± standard deviation. *n* = 5 or 6, **p < .01 versus control, as calculated by using the Student's *t*‐test

Next, we fractionated the CH_2_Cl_2_ extract, which is known to have unidentified biofunctional molecules, with hexane:EtOAc = 10:0, 8:2, 6:4, 4:6, 2:8, and 0:10 and MeOH using silica gel column chromatography (Figure 2a). We then assessed these fractions using ZOT and found that Fr. 2 significantly (*p* < .05) reduced the amount of VAT compared to the control treatment (Figure 2b,c). The results of testing for reproducibility are presented in Figure S4.

To elucidate the anti‐obesity mechanisms of Fr. 2, we examined the expression of genes involved in adipocyte differentiation in the VAT of young zebrafish. Expression of early adipogenesis markers, *cebpb* and *cebpd*, was significantly (*p* < .05) decreased by Fr. 2 (Figure 2d), as was the expression of the late differentiation marker *cebpa* (Figure 2e).

### 3.3 Subfractions of Fr. 2 suppressed adipogenesis

**FIGURE 3:**
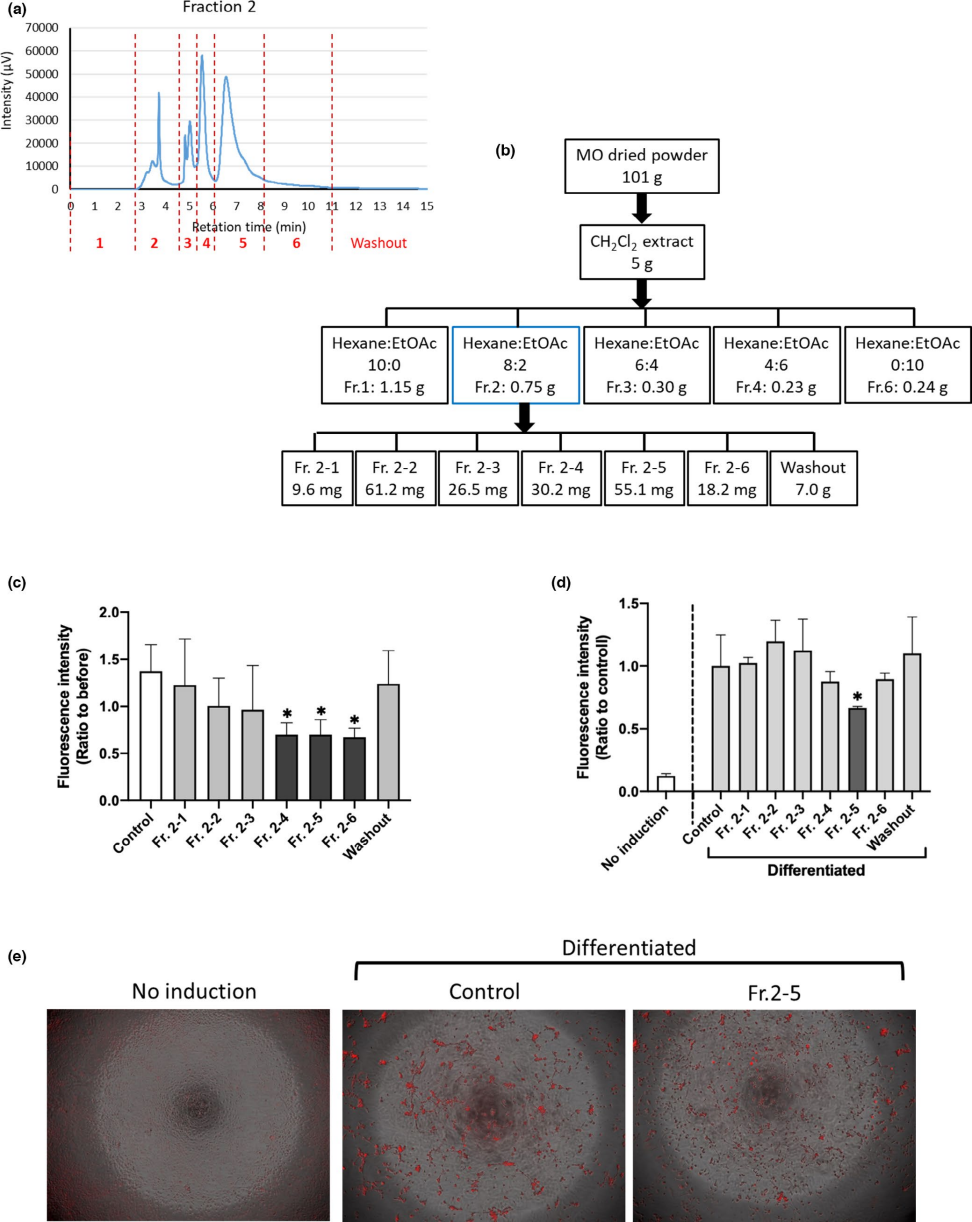
Subfractions of Fr. 2 suppress adipogenesis. (a) Fr. 2 was subfractionated into seven pools including washout. (b) The yield of each step. (c) Zebrafish obesogenic test. the 4th, 5th, and 6th fractions (10 μg/ml) reduced the amount of VAT. *p < .05 versus control, *n* = 5, error bars indicate standard deviation (*SD*). (d) In vitro adipogenesis assays using mouse preadipocyte 3T3‐L1 cells. Data are shown as the means ± *SD*. *n* = 8, *p < .05 versus control, as calculated by using one‐way ANOVA. (e) Representative images of (d). Red indicates mature adipocytes

To fractionate Fr. 2, we increased the MO input to 101 g and divided the Fr. 2 into seven fractions (including washout) using preparative HPLC (Figure 3a), and the dried yield of each subfraction is presented in Figure 3b. Each subfraction was analyzed using preparative HPLC to confirm that the peaks were separated (Figure S5). We then performed ZOT with these seven fractions and found that the 4th, 5th, and 6th fractions significantly (*p* < .05) reduced VAT accumulation compared to the control treatment (Figure 3c). Although we have already demonstrated that ZOT results are comparable to mouse 3T3‐L1 preadipocyte differentiation experiment results (Nakayama et al., 2020), we performed a 3T3‐L1 cell‐based adipogenesis assay with these fractions. As shown in Figure 3d, the 5th fraction of the Fr. 2, denoted as Fr. 2–5, significantly (*p* < .05) suppressed lipid accumulation in the cells. Representative images are shown in Figure 3e.

## 4 DISCUSSION

In this study, we showed for the first time that the CH_2_Cl_2_ extract (10 μg/ml) of MO suppressed adipogenesis, equivalent to 10 μM phenylephrine treatment (Figure 1c,d). MO leaf aqueous, MeOH, and EtOH extracts have been shown to have anti‐obesity and anti‐lipidemic effects in rodents (Ezzat et al., 2020; Huang et al., 2020; Kilany et al., 2020; Kim et al., 2020). We have summarized the comparison between our findings and previously identified bioactive compounds in MO (Table 1). Of these, Liao et al. identified beta‐sitosterol in MeOH extracts of MO (Liao et al., 2018), which protects against high‐fructose diet‐induced metabolic dysfunction in rats (Gumede et al., 2020). We also performed ZOT with beta‐sitosterol (10 μg/ml) and found that it did not significantly suppress VAT accumulation (Figure S6). MeOH extract also did not affect VAT in zebrafish (Figure 1c). It is difficult to determine the reasons for these differences, although there is a possibility that differences between zebrafish and mammals in the digestive system or metabolism are involved in the anti‐adipogenic response. To identify the molecules responsible for the activity of the Fr. 2–5, we performed NMR analysis; however, we did not have sufficient purity (data not shown). This subfraction contains multiple constituents, suggesting that a mixture of bioactive molecules in Fr. 2–5 exhibited anti‐adipogenic effects. Further fractionation using large‐scale extraction is necessary to identify the individual molecules.

**TABLE 1:** Bioactive compounds in MO

Name	Efficacy	Reference	Possibility to contain in the CH_2_Cl_2_ extract
Beta‐sitosterol	Protective for metabolic dysfunction in high‐fructose diet‐induced rat. Not effective in ZOT in our study (Figure S6).	Gumede et al. (2020)	Yes
Epicatechin‐3‐galloyl ester (ECG)	Green tea extract containing ECG reduces VAT in obese mouse and zebrafish.	Cunha et al. (2013), Zang et al. (2019)	Possibly yes
Chlorogenic acid	Improve lipid and glucose metabolism in obese Zucker rats.	Rodriguez de Sotillo et al. (2002)	Possibly yes
Astragalin	3‐O‐glucoside of kaempferol. Inhibit adipogenesis in 3T3‐L1 adipocytes.	Swamy et al. (2020)	No
Isothiocyanates	Inhibit adipogenesis in 3T3‐L1 adipocytes.	Huang et al. (2020)	Yes

Although it is difficult to compare the adipogenic timelines between ZOT and mammals, Fr. 2 downregulated the expression of early adipogenesis markers, namely, *cebpb* and *cebpd*, which regulate the expression of the late adipogenesis markers *pparg* and *cebpa* (Figure 2d,e), indicating that Fr. 2 suppresses early‐stage adipogenesis. Since the adipogenic transcriptome profiles under conditions that promote VAT accumulation are common between adult zebrafish and mice (Nakayama et al., 2018; Y. Shimada et al., 2014), our identified mechanism of Fr. 2 activity exists in vertebrates. In addition, several flavonoids have been reported to suppress early adipogenesis. For example, kaempferol suppresses early‐stage adipogenesis by downregulating *cebpb* expression in zebrafish and 3T3‐L1 cells (Lee et al., 2015), similar to that seen in our study. The kaempferol derivative astragalin has been found in MO extract in previous studies (Lin et al., 2019; Swamy et al., 2020).

Cultured adipocytes and mouse models have usually been used for screening obesity‐suppressing compounds. Because cultured adipocytes reflect a small part of the biological processes that take place in living organisms, the results of cell‐based studies could not always be reproduced in whole‐animal studies. In addition, it is difficult to predict the side effects of the tested compounds in cell cultures (Ghanemi, 2015). The information obtained from rodent studies is relatively similar to that obtained from human studies, making several researchers believe that rodents are ideal animal models. However, it takes several months to complete the test using a HFD rodent obesity model, making it difficult to test a large number of compounds. In addition, from the standpoint of animal welfare, it is also difficult to use rodents for screening studies.

The present technique using juvenile zebrafish, ZOT, could reduce the time (less than 1 week, which is shorter than that of 3T3‐L1 adipocyte testing), cost, and amount of the tested compounds (almost the same as the 3T3‐L1 testing). In addition to the efficacy evaluation, ZOT could also be used to detect toxicity in whole‐animal studies. Moreover, we found that a high concentration (100 μg/ml) of MO extracts or fractions killed zebrafish (Figure S2).

At the endpoint of the ZOT, we collected NR‐labelled (yellow fluorescent) adipose tissue by pipetting under fluorescent microscopy. Because transcriptome analysis can be performed with a small portion of RNA samples, ZOT has a great advantage in gene expression analysis with this simple tissue collection method, in addition to the throughput and phenotypic analysis that we previously summarized with advantages and limitations (Nakayama et al., 2020). Proteome analysis requires a relatively large amount of tissue that is unaffordable in juvenile zebrafish, and hence, adult fish experiments are recommended.

## 5 CONCLUSION

Using ZOT, we identified that a CH_2_Cl_2_ extract of MO and Fr. 2 obtained using an extract fractionation procedure suppressed VAT accumulation. Fr. 2 downregulated the expression of genes responsible for early adipogenesis, thereby suppressing adipogenesis in zebrafish VAT. We demonstrated that the anti‐adipogenic subfractions of Fr. 2 were also effective in 3T3‐L1 cell‐based assays. In addition to known anti‐obesity molecules in aqueous MO extract, we expect multiple constituents in non‐aqueous extracts to reduce visceral adiposity. The ZOT using juvenile fish can be applied for phenotype‐driven screening to discover bioactive constituents with gene expression analysis in fluorescent‐labelled VAT.

## CONFLICT OF INTEREST

Youngil Kim, Djong‐Chi Chu, and Lekh Raj Juneja are employees of Rohto Pharmaceutical Co., Ltd.. The other authors declare no competing interests.

## ETHICAL APPROVAL

All animal procedures were approved by the Ethics Committee of Mie University, Tsu, Japan (Permit Number 28‐4‐1). Animal experiments were performed in accordance with the Japanese Welfare Regulatory Practice Act on Welfare and Management of Animals (Ministry of Environment of Japan) and complied with international guidelines.

## Supporting information

Fig S1‐S6

TableS1
