# Supplementary material for: Zebrafish obesogenic test identifies anti‐adipogenic fraction in Moringa oreifera leaf extracts
Source: Food Sci Nutr. 2022 Feb 11;10(4):1248–56. doi: 10.1002/fsn3.2758 (PMC9007296; doi:10.1002/fsn3.2758)
Supplement: Supplementary file 1 — Fig S1‐S6 [file FSN3-10-1248-s001.docx]

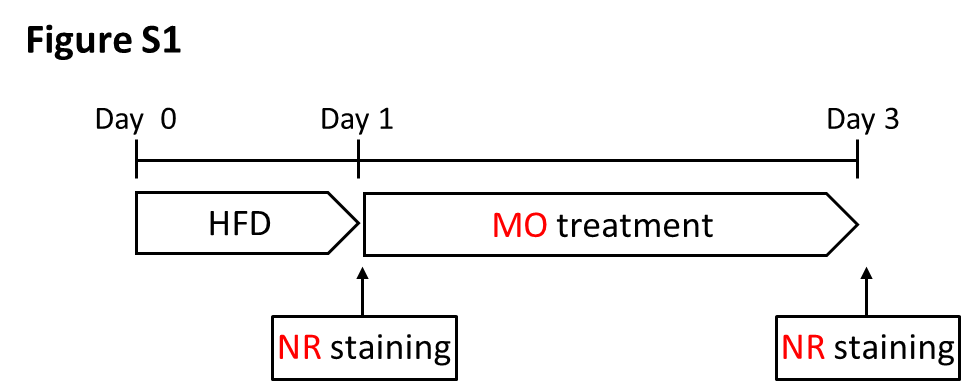


**Figure S1. ZOT experimental design.**

**
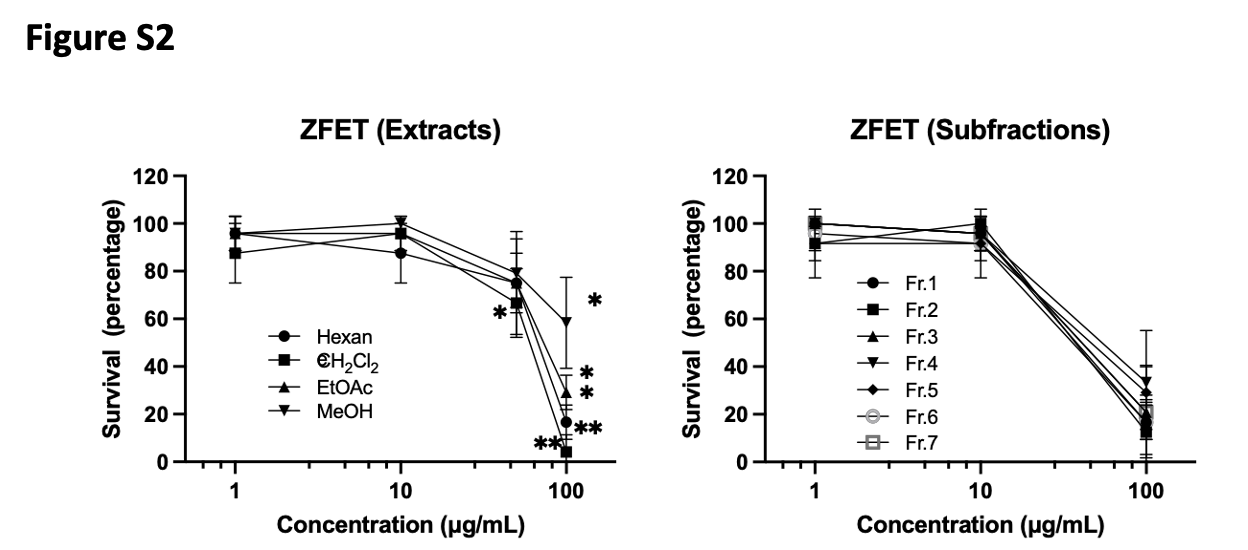
**

**Figure S2. Zebrafish embryonic acute toxicity test (ZFET) was conducted to decide safety concentrations.** Each extract or subtraction was added to the embryos in the blastula stage (approximately 5 h post fertilisation (hpf)), and the surviving embryos were counted after 96 hpf. Each group contained eight fish with three triplicates. **p* < 0.05 and ***p* < 0.01 vs. control (0 μg/mL concentration), error bars indicate SD.

**
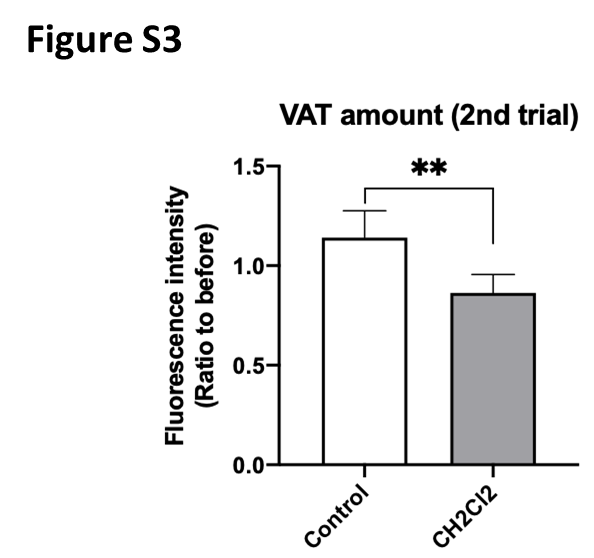
**

**Figure S3. Repeat ZOT ~~trial~~ for CH_2_Cl_2_ extract efficacy.** NR fluorescence intensities in VAT. The Y-axis indicates the ratio of NR staining before and after treatment for 48 h with extract. n = 5, ***p* < 0.01 vs. control, error bars indicate SD.


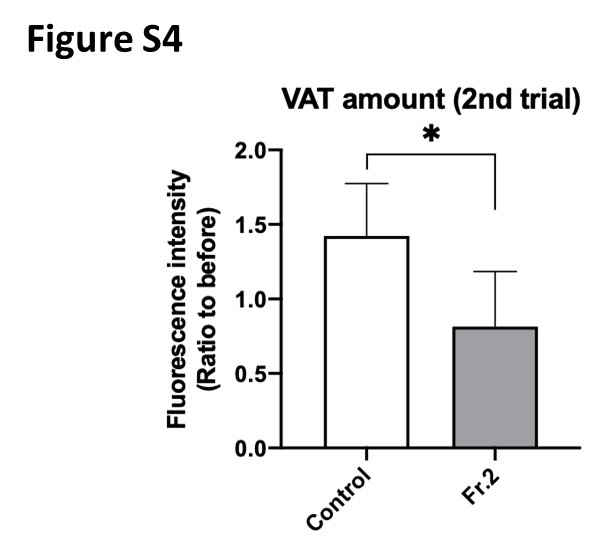


**Figure S4. Second ZOT ~~trial~~ assessing Fr. 2 efficacy.** Zebrafish obesogenic test for Fr. 2. The Y-axis indicates the ratio of NR staining before and after treatment for 48 h with Fr. 2. n = 5, **p* < 0.05 vs. control, error bars indicate SD.


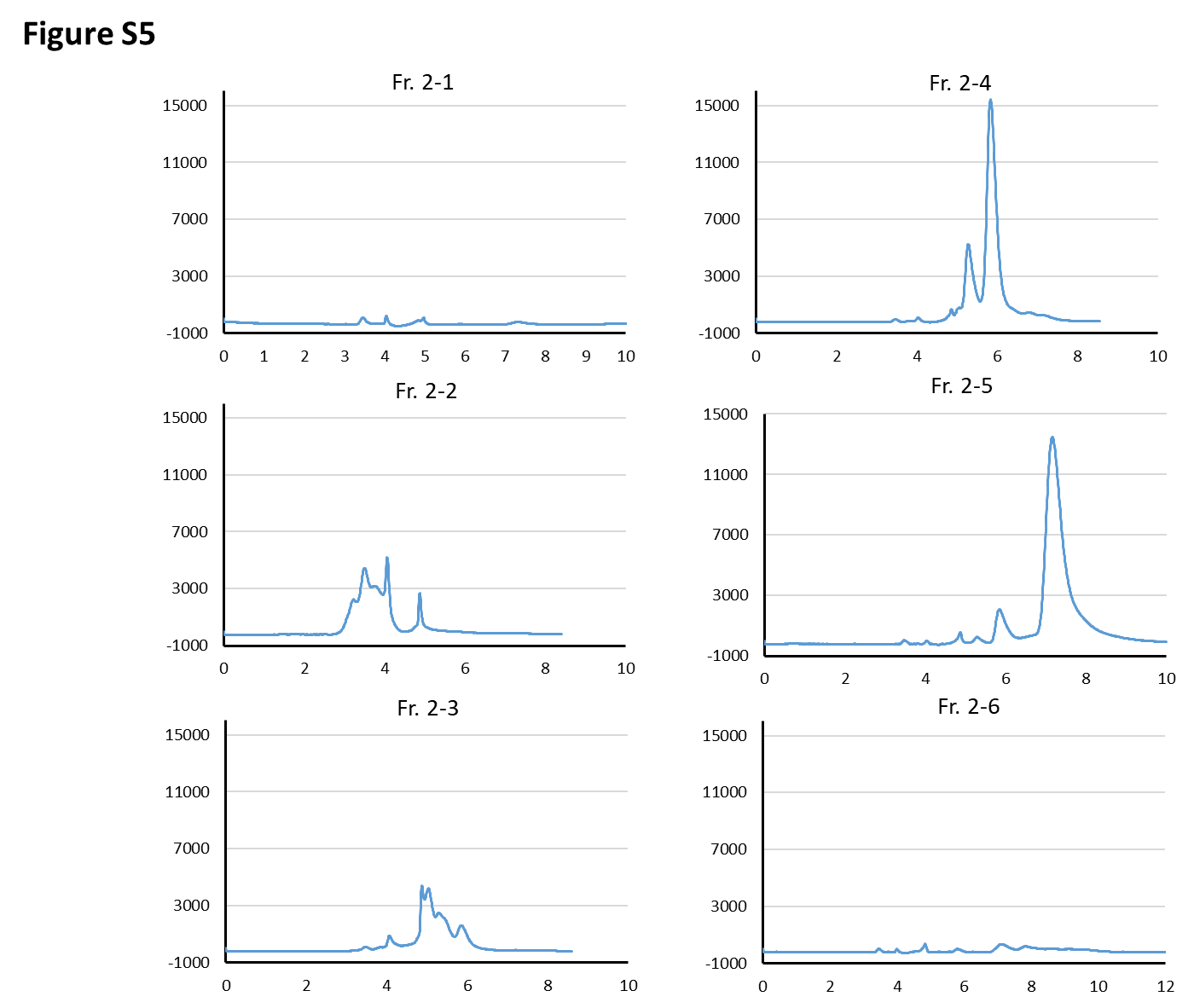


**Figure S5. RP-HPLC chromatogram for each subfraction of Fr. 2.**


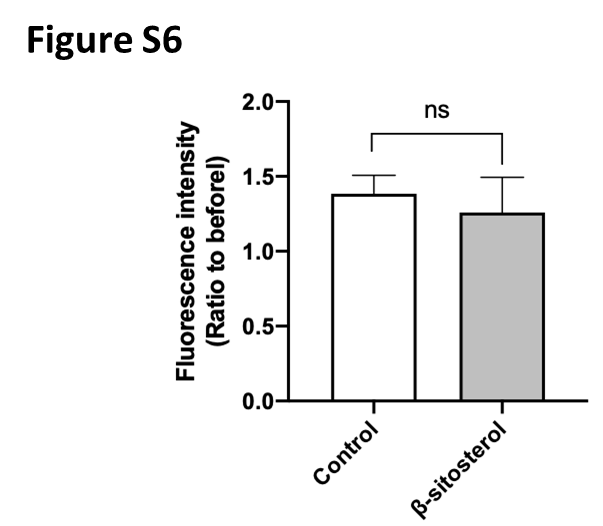


**Figure S6. ZOT for β~~beta~~-sitosterol.** The Y-axis indicates the ratio of NR staining before and after treatment with β~~beta~~-sitosterol for 48 h. n = 5, ns indicates no significant. Error bars indicate SD.
